# Supplementary material for: The indirect impact of COVID-19 pandemic on inpatient admissions in 204 Kenyan hospitals: An interrupted time series analysis
Source: PLOS Glob Public Health. 2021 Nov 17;1(11):e0000029. doi: 10.1371/journal.pgph.0000029 (PMC10021711; doi:10.1371/journal.pgph.0000029)
Supplement: S1 File — The definition of the indicators analysed is outlined. (DOCX) [file pgph.0000029.s001.docx]

**Supplementary Information 1**

**Fig 1 Distribution of the 204 hospitals in each county**


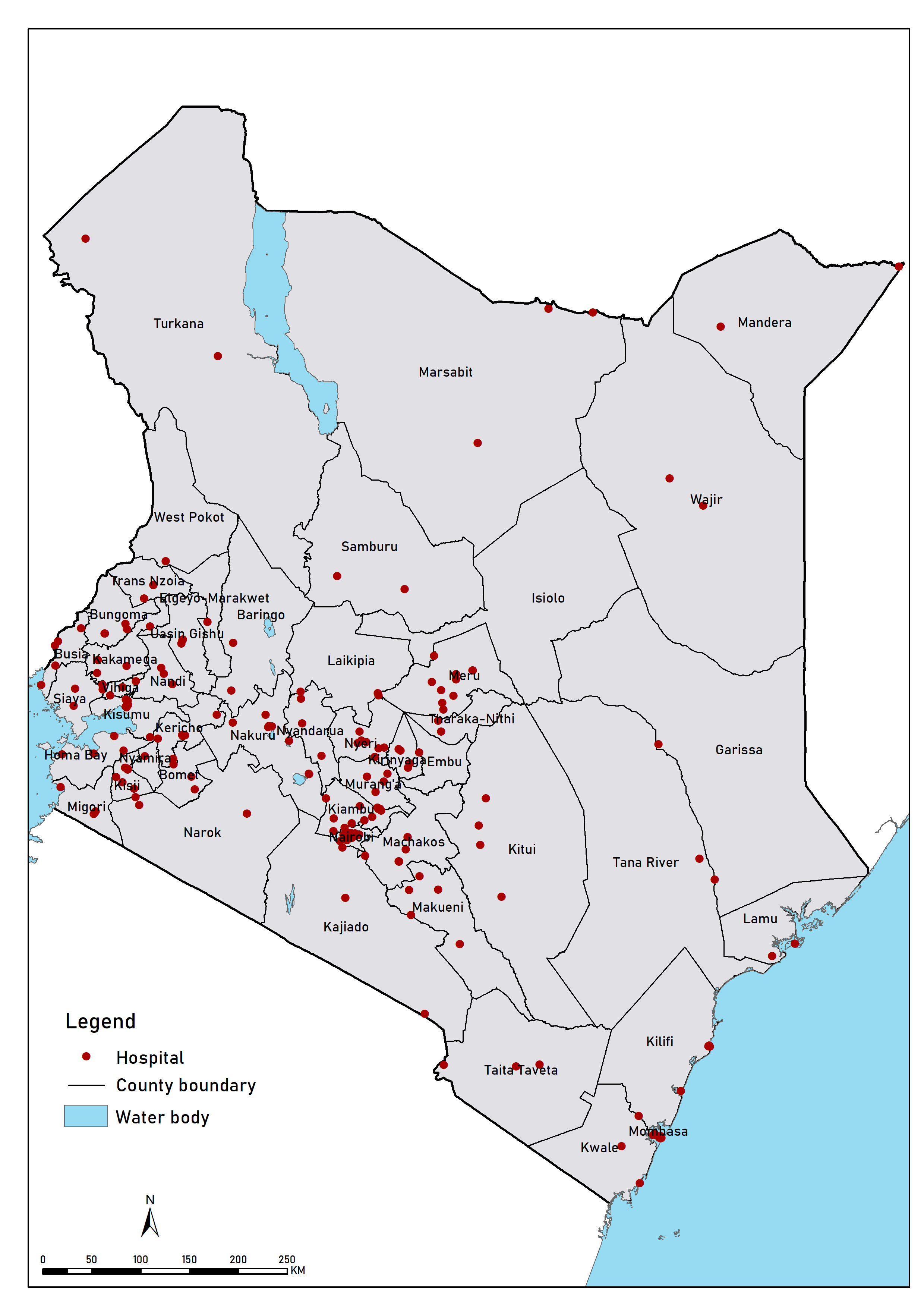


**Table 1: Indicators used to select service availability at hospitals from the EMFK, HHFA and SARAM. The orange areas are those needed for VLBW service, green are those needed for CS services and blue are those needed for both tracer conditions.**

| **Indicator** | **SARAM (2013)** | **EMFK (2018)** | **HHFA (2018)** |
| --- | --- | --- | --- |
| Availability of at least two registered nurses, who presumably provide critical KMC. KMC only available in HHFA | Reports number of nurses in each facility. Includes a separate category of registered nurses | No data | Reports number of nurses in each facility. Includes a separate category of registered nurses |
| Availability of at least two medical officers | Reports the number of medical officers in each facility. | Reports whether there are medical officers providing emergency surgery | Reports the number of medical officers in each facility. |
| Chest x-ray services offered | Whether available or not | No data | Whether available only. No unavailability data |
| Intravenous fluids (IV fluids) available | Whether available only. No unavailability data | No data | Whether available or not |
| Oxygen Services | Whether available or not | No data | Whether available or not |
| Infant incubator | Whether available or not | No data | Whether available or not |
| Operating theatre | Whether available or not | Whether available or not | Whether available or not |
| Blood transfusion services | Whether available or not | Whether available or not | Whether available or not |
| Availability of basic surgical equipment. Includes a theatre bed, instruments for incision (forceps, needle driver and sterile scissors), anaesthetic machine and infection prevention control. | Whether available only. No unavailability data | No data | Whether available only. No unavailability data |

**Table 2: Distribution of 204 hospitals by level and ownership**

|  |  | Ownership | | |  |
| --- | --- | --- | --- | --- | --- |
|  |  | **Public** | **FBO** | **Private** | **Total** |
| Level | **Level 3** | 0 | 0 | 2 | **2** |
|  | **Level 4** | 94 | 41 | 47 | **182** |
|  | **Level 5** | 13 | 3 | 2 | **18** |
|  | **Level 6** | 2 | 0 | 0 | **2** |
|  | **Total** | **109** | **44** | **51** | **204** |

**Footnote:**

**Levels description**

Level 3: Comprehensive primary health care facility

Level 4: Primary referral hospitals

Level 5: Secondary referral hospitals

Level 6: National teaching and referral hospitals)

**Ownership**

MoH – Ministry of Health (Public)

FBO – Faith Based Organization

Private – Hospitals that are neither MoH or FBO

NB: Faith Based Organizations (FBO) and Private were combined into private because they both belong to private entities and hence similar.

**Table 3: Definition of indicators**

| **Indicator** | **Source Form** | **Definition** |
| --- | --- | --- |
| Admissions > 5 Medical | MOH 717 | Number of patients over 5 years admitted in medical ward |
| Admissions > 5 Surgical | MOH 717 | Number of patients over 5 years admitted in surgical ward |
| Admissions < 5 Paediatrics | MOH 717 | Number of patients under 5 years admitted in paediatrics ward |
| NBU Admissions | MOH 717 | Number of patients under 5 years admitted in nursery/new-born unit ward |
| Total deliveries | MOH 711 | Number of deliveries conducted in Maternity |
| Caesarean Sections | MOH 711 | Number of caesarean sections conducted in Maternity |
| Live births | MOH 711 | Number of live births in Maternity |

**Table 4: Number and percentage of hospitals analysed. There was 109 public and 95 private hospitals**

| **Public Hospitals** | | | | | |
| --- | --- | --- | --- | --- | --- |
| **Indicator** | **All hospitals** | **Hospitals not reporting any month** | **Hospitals reporting at least a month** | **Number of Imputed/analysed hospitals** | **Percentage of hospitals analysed out of hospitals reporting at least a month** |
| Caesarian sections | 109 | 2 | 107 | 106 | 99.1 |
| Admissions > 5 Medical | 109 | 5 | 104 | 102 | 98.1 |
| Admissions > 5 Surgical | 109 | 52 | 57 | 48 | 84.2 |
| Admissions NBU | 109 | 36 | 73 | 61 | 83.6 |
| Admissions < 5 Paediatrics | 109 | 3 | 106 | 100 | 94.3 |
| Admissions > 5 Maternity | 109 | 3 | 106 | 102 | 96.2 |
| Live Births | 109 | 1 | 108 | 108 | 100.0 |
| Total deliveries | 109 | 1 | 108 | 108 | 100.0 |
| **Private Hospitals** | | | | | |
| Caesarian sections | 95 | 1 | 94 | 93 | 98.9 |
| Admissions > 5 Medical | 95 | 3 | 92 | 89 | 96.7 |
| Admissions > 5 Surgical | 95 | 13 | 82 | 67 | 81.7 |
| Admissions NBU | 95 | 35 | 60 | 32 | 53.3 |
| Admissions < 5 Paediatrics | 95 | 4 | 91 | 83 | 91.2 |
| Admissions > 5 Maternity | 95 | 3 | 92 | 86 | 93.5 |
| Live Births | 95 | 0 | 95 | 95 | 100.0 |
| Total deliveries | 95 | 0 | 95 | 95 | 100.0 |

**Number of hospitals imputed** – Hospitals reporting at least 11/39 (30%) of months

**Hospitals reporting at least a month** – All hospitals ***minus*** Hospitals not reporting any month over the period of study
