# Supplementary material for: The indirect impact of COVID-19 pandemic on inpatient admissions in 204 Kenyan hospitals: An interrupted time series analysis
Source: PLOS Glob Public Health. 2021 Nov 17;1(11):e0000029. doi: 10.1371/journal.pgph.0000029 (PMC10021711; doi:10.1371/journal.pgph.0000029)
Supplement: S2 File — (DOCX) [file pgph.0000029.s002.docx]

**Figure 1: Missing data patterns plot showing number of reported months by hospitals. The x – axis shows the number of months reported by health facilities. 0 means the hospitals did not report any month, while 39 means the hospitals reported all months.**


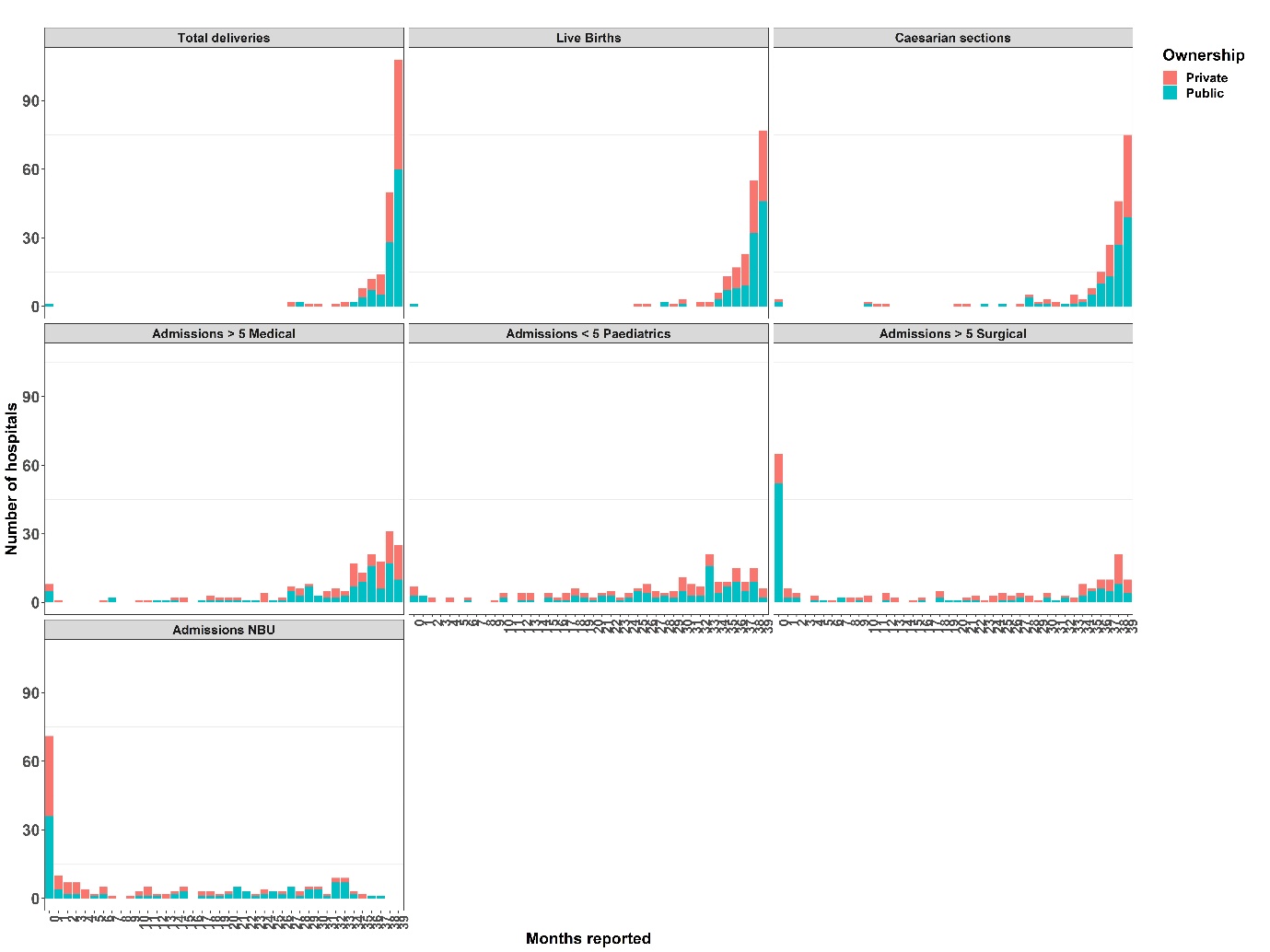


**Figure 2: Multiple change points for public hospitals Red boxes show where services changed due to the national health care workers strike (December 2020)**

**
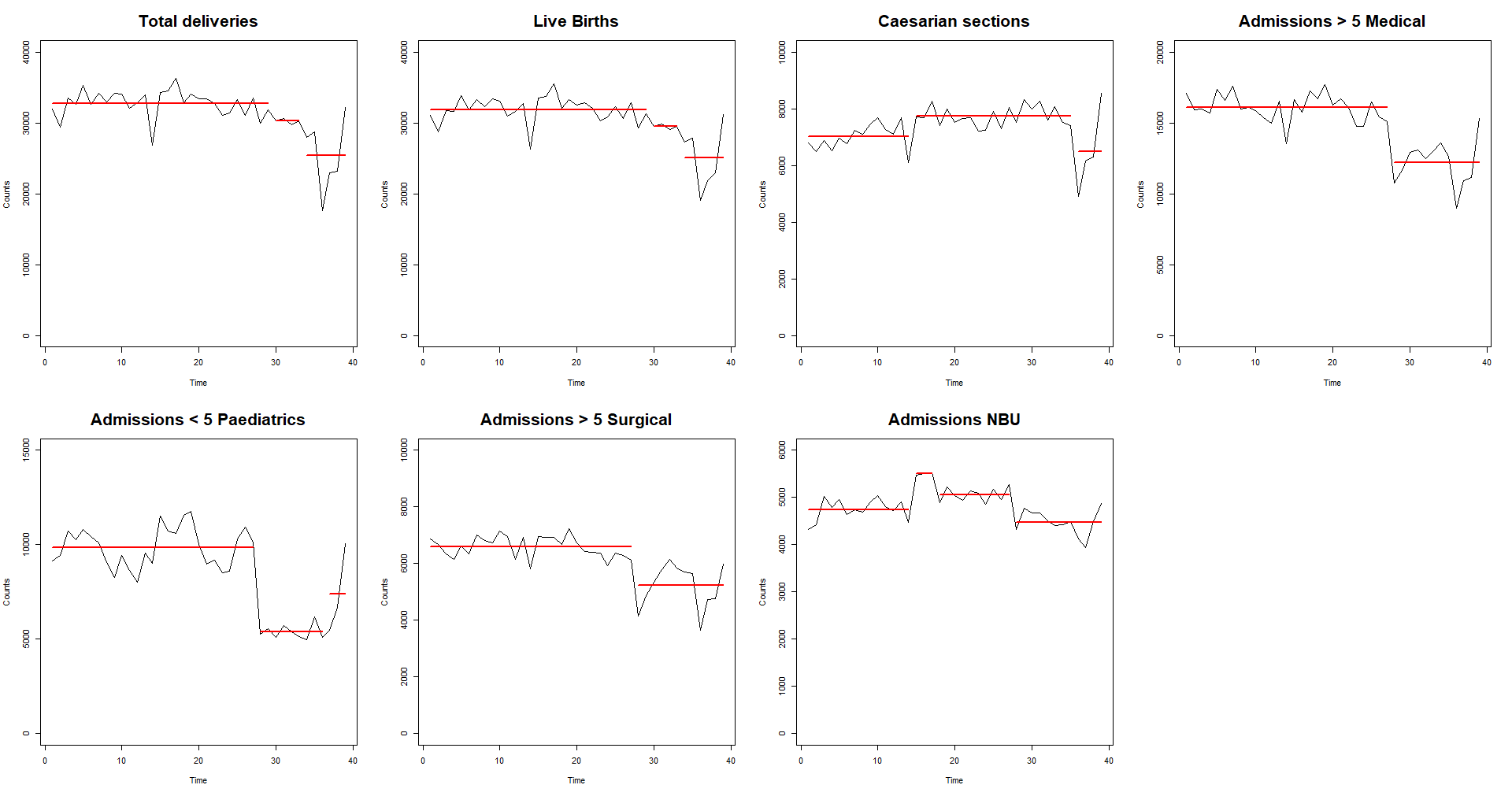
**

Nov20

**Figure 3: Multiple change points for private hospitals.** **Red boxes show where services changed due to the national health care workers strike (December 2020)**

**
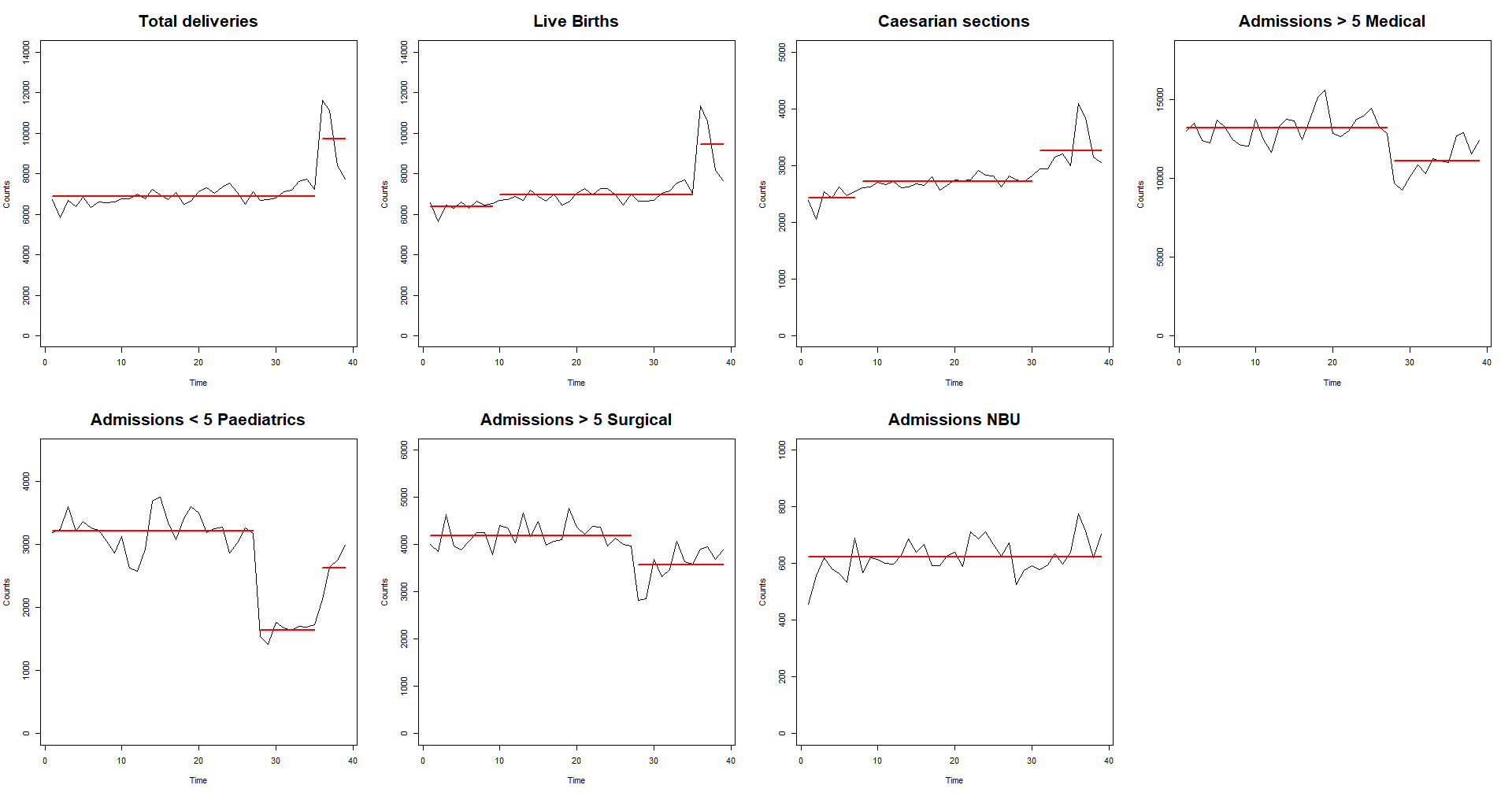
**

Nov20

Nov20
