# Supplementary material for: The indirect impact of COVID-19 pandemic on inpatient admissions in 204 Kenyan hospitals: An interrupted time series analysis
Source: PLOS Glob Public Health. 2021 Nov 17;1(11):e0000029. doi: 10.1371/journal.pgph.0000029 (PMC10021711; doi:10.1371/journal.pgph.0000029)
Supplement: S3 File — (DOCX) [file pgph.0000029.s003.docx]

**Table 1: Interrupted time series results excluding months affected by national strike (December 2020 – February 2021) showing incidence rate ratios (RR) and relative risk (RR) for COVID-19 intervention, time and trend alongside 95% confidence intervals – Public Hospitals**

|  |  | **Total deliveries** | | | **Live births** | | | **Caesarean sections rate** | | | **Admissions > 5 Medical** | | |
| --- | --- | --- | --- | --- | --- | --- | --- | --- | --- | --- | --- | --- | --- |
| **Model** |  | **(IRR** | **95%CI** | **P-value** | **(IRR** | **95%CI** | **P-value** | **RR** | **95%CI** | **P-value** | **(IRR** | **95%CI** | **P-value** |
| **Primary model** | **COVID-19** | 0.89 | (0.79-1.02) | 0.08 | 0.89 | (0.80-0.99) | 0.04 | 1.10 | (1.06-1.14) | <0.01 | 0.71 | (0.63-0.79) | <0.01 |
|  | **Time** | 1.00 | (0.99-1.01) | 0.67 | 1.00 | (0.99-1.01) | 0.42 | 1.01 | (1.00-1.01) | <0.01 | 1.00 | (0.99-1.00) | 0.57 |
|  | **Trend** | 0.99 | (0.97-1.00) | 0.11 | 0.99 | (0.97-1.00) | 0.07 | 1.00 | 0.99-1.00) | 0.14 | 1.01 | (0.99-1.03) | 0.10 |
| **Excluding Strike** | **COVID-19** | 0.87 | (0.80-0.95) | <0.01 | 0.87 | (0.80-0.94) | <0.01 | 1.11 | (1.06-1.16) | <0.01 | 0.65 | (0.62-0.69) | <0.01 |
|  | **Time** | 1.00 | (0.98-1.00) | 0.62 | 1.00 | (0.99-1.00) | 0.30 | 1.01 | (1.00-1.01) | <0.01 | 1.00 | (0.99-1.00) | 0.33 |
|  | **Trend** | 1.01 | (0.99-1.02) | 0.42 | 1.00 | (0.99-1.02) | 0.52 | 0.99 | (0.99-1.00) | 0.07 | 1.04 | (1.03-1.05) | <0.01 |
|  | | | | | | | | | | | | | |
|  |  | **Admissions < 5 Paediatrics** | | | **Admissions > 5 Surgical** | | | **NBU Admissions rate** | | |  | | |
| **Model** |  | **(IRR** | **95%CI** | **P-value** | **(IRR** | **95%CI** | **P-value** | **RR** | **95%CI** | **P-value** |  |  |  |
| **Primary model** | **COVID-19** | 0.41 | (0.38-0.45) | <0.01 | 0.75 | (0.64-0.87) | <0.01 | 0.92 | (0.83-1.01) | 0.09 |  |  |  |
|  | **Time** | 1.00 | (1.00-1.01) | <0.01 | 1.00 | (0.99-1.00) | 0.46 | 1.00 | (1.00-1.01) | 0.02 |  |  |  |
|  | **Trend** | 1.04 | (1.03-1.05) | <0.01 | 1.01 | (0.99-1.03) | 0.19 | 1.01 | (1.00-1.02) | 0.04 |  |  |  |
| **Excluding Strike** | **COVID-19** | 0.37 | (0.32-0.42) | <0.01 | 0.71 | (0.64-0.79) | <0.01 | 0.92 | (0.86-0.99) | 0.03 |  |  |  |
|  | **Time** | 1.00 | (1.00-1.01) | 0.01 | 1.00 | (0.99-1.00) | 0.21 | 1.00 | (1.00-1.01) | <0.01 |  |  |  |
|  | **Trend** | 1.08 | (1.06-1.11) | <0.01 | 1.04 | (1.02-1.05) | <0.01 | 1.00 | (0.99-1.02) | 0.47 |  |  |  |

**Table 2: Interrupted time series results excluding months affected by national strike (December 2020 – February 2021) showing incidence rate ratios (IRR) and relative risk (RR) for COVID-19 intervention, time and trend alongside 95% confidence intervals – Private Hospitals**

|  |  | **Total deliveries** | | | **Live births** | | | **Caesarian sections rate** | | | **Admissions > 5 Medical** | | |
| --- | --- | --- | --- | --- | --- | --- | --- | --- | --- | --- | --- | --- | --- |
| **Model** |  | **IRR** | **95%CI** | **P-value** | **IRR** | **95%CI** | **P-value** | **RR** | **95%CI** | **P-value** | **IRR** | **95%CI** | **P-value** |
| **Primary model** | **COVID-19** | 0.91 | (0.81-1.03) | 0.13 | 0.91 | (0.81-1.03) | 0.12 | 1.03 | (0.98-1.09) | 0.25 | 0.66 | (0.60-0.72) | <0.01 |
|  | **Time** | 1.00 | (0.99-1.01) | 0.06 | 1.00 | (0.99-1.01) | 0.03 | 1.00 | (1.00-1.01) | 0.02 | 1.00 | (1.00-1.01) | 0.01 |
|  | **Trend** | 1.03 | (1.01-1.04) | <0.01 | 1.03 | (1.01-1.04) | <0.01 | 0.99 | (0.98-0.99) | 0.01 | 1.03 | (1.01-1.04) | <0.01 |
| **Excluding Strikes** | **COVID-19** | 0.94 | (0.87-1.00) | 0.06 | 0.93 | (0.87-1.00) | 0.07 | 1.02 | (0.97-1.07) | 0.53 | 0.64 | (0.56-0.72) | <0.01 |
|  | **Time** | 1.00 | (1.00-1.01) | <0.01 | 1.00 | (1.00-1.01) | <0.01 | 1.00 | (1.00-1.01) | <0.01 | 1.00 | (1.00-1.01) | 0.01 |
|  | **Trend** | 1.01 | (1.00-1.02) | 0.09 | 1.01 | (0.99-1.02) | 0.14 | 1.00 | (0.99-1.01) | 0.74 | 1.03 | (1.01-1.05) | <0.01 |
|  | | | | | | | | | | | | | |
|  |  | **Admissions < 5 Paediatrics** | | | **Admissions > 5 Surgical** | | | **NBU Admissions rate** | | |  | | |
| **Model** |  | **IRR** | **95%CI** | **P-value** | **IRR** | **95%CI** | **P-value** | **RR** | **95%CI** | **P-value** |  |  |  |
| **Primary model** | **COVID-19** | 0.38 | (0.33-0.45) | <0.01 | 0.72 | (0.64-0.81) | <0.01 | 0.87 | (0.72-1.04) | 0.13 |  |  |  |
|  | **Time** | 1.00 | (0.99-1.01) | 0.38 | 1.00 | (0.99-1.00) | 0.82 | 1.00 | (0.99-1.01) | 0.21 |  |  |  |
|  | **Trend** | 1.07 | (1.05-1.08) | <0.01 | 1.02 | (1.01-1.04) | <0.01 | 0.99 | (0.97-1.02) | 0.48 |  |  |  |
| **Excluding Strikes** | **COVID-19** | 0.36 | (0.30-0.44) | <0.01 | 0.69 | (0.61-0.8) | <0.01 | 0.83 | (0.68-1.01) | 0.07 |  |  |  |
|  | **Time** | 1.00 | (1.00-1.01) | 0.37 | 1.00 | (0.99-1.00) | 0.74 | 1.00 | (1.00-1.01) | 0.20 |  |  |  |
|  | **Trend** | 1.08 | (1.05-1.11) | <0.01 | 1.03 | (1.01-1.05) | <0.01 | 1.01 | (0.98-1.04) | 0.56 |  |  |  |

**Table 3: Intraclass correlation coefficient (ICC)**

| **Indicator** | **All hospitals (Public and Private)** | **Public** | **Private** |
| --- | --- | --- | --- |
|  | **ICC** | **ICC** | **ICC** |
| **Total deliveries** | 0.91 | 0.88 | 0.83 |
| **Live births** | 0.91 | 0.89 | 0.84 |
| **Caesarian sections** | 0.90 | 0.90 | 0.86 |
| **Admissions > 5 Medical** | 0.79 | 0.82 | 0.77 |
| **Admissions > Surgical** | 0.86 | 0.89 | 0.71 |
| **Admissions < 5 Paediatrics** | 0.74 | 0.72 | 0.56 |
| **NBU admissions** | 0.92 | 0.89 | 0.92 |

**Table 4: Generalised estimating equations (GEE) results at hospital level showing incidence rate ratios (IRR) for COVID-19 intervention, time and trend alongside 95% confidence intervals for all indicators**

|  |  | **Total deliveries** | | | **Live births** | | | **Caesarean sections rate** | | | **Admissions > 5 Medical** | | |
| --- | --- | --- | --- | --- | --- | --- | --- | --- | --- | --- | --- | --- | --- |
| **Ownership** |  | **IRR** | **95%CI** | **P-value** | **IRR** | **95%CI** | **P-value** | **IRR** | **95%CI** | **P-value** | **IRR** | **95%CI** | **P-value** |
| **Public** | **COVID-19** | 0.90 | (0.83-0.97) | 0.01 | 0.89 | (0.82-0.97) | 0.01 | 1.12 | (1.06-1.14) | <0.01 | 0.70 | (0.65-0.76) | <0.01 |
|  | **Time** | 1.00 | (0.99-1.00) | 0.32 | 1.00 | (0.99-1.00) | 0.11 | 1.01 | (1.00-1.01) | <0.01 | 1.00 | (0.99-1.00) | 0.40 |
|  | **Trend** | 0.99 | (0.98-0.99) | <0.01 | 0.99 | (0.98-0.99) | 0.01 | 1.00 | 0.98-1.00) | 0.09 | 1.01 | (1.01-1.02) | <0.01 |
| **Private** | **COVID-19** | 0.92 | (0.86-0.98) | 0.01 | 0.91 | (0.86-0.98) | 0.01 | 1.03 | (0.98-1.08) | 0.16 | 0.66 | (0.60-0.74) | <0.01 |
|  | **Time** | 1.00 | (0.99-1.01) | 0.07 | 1.00 | (1.00-1.01) | 0.04 | 1.01 | (1.00-1.01) | <0.01 | 1.00 | (0.99-1.01) | 0.07 |
|  | **Trend** | 1.03 | (1.02-1.04) | <0.01 | 1.03 | (1.02-1.04) | <0.01 | 1.02 | (1.01-1.03) | <0.01 | 1.02 | (1.01-1.04) | <0.01 |
|  | | | | | | | | | | | | | |
|  |  | **Admissions < 5 Paediatrics** | | | **Admissions > 5 Surgical** | | | **NBU Admissions rate** | | |  | | |
| **Ownership** |  | **IRR** | **95%CI** | **P-value** | **IRR** | **95%CI** | **P-value** | **IRR** | **95%CI** | **P-value** |  |  |  |
| **Public** | **COVID-19** | 0.41 | (0.37-0.45) | <0.01 | 0.77 | (0.69-0.85) | <0.01 | 0.87 | (0.78-1.01) | 0.08 |  |  |  |
|  | **Time** | 1.00 | (1.00-1.01) | 0.03 | 1.00 | (0.99-1.00) | 0.37 | 1.01 | (1.00-1.01) | <0.01 |  |  |  |
|  | **Trend** | 1.05 | (1.03-1.07) | <0.01 | 1.01 | (0.99-1.03) | 0.26 | 1.00 | (0.99-1.01) | 0.84 |  |  |  |
| **Private** | **COVID-19** | 0.38 | (0.32-0.45) | <0.01 | 0.72 | (0.62-0.85) | <0.01 | 0.84 | (0.79-1.02) | 0.07 |  |  |  |
|  | **Time** | 1.00 | (0.99-1.01) | 0.51 | 1.00 | (0.99-1.01) | 0.86 | 1.01 | (0.99-1.02) | 0.06 |  |  |  |
|  | **Trend** | 1.07 | (1.05-1.09) | <0.01 | 1.02 | (1.01-1.04) | <0.01 | 1.02 | (0.99-1.04) | 0.22 |  |  |  |
