# Supplementary material for: The indirect impact of COVID-19 pandemic on inpatient admissions in 204 Kenyan hospitals: An interrupted time series analysis
Source: PLOS Glob Public Health. 2021 Nov 17;1(11):e0000029. doi: 10.1371/journal.pgph.0000029 (PMC10021711; doi:10.1371/journal.pgph.0000029)
Supplement: S4 File — (DOCX) [file pgph.0000029.s004.docx]

**Supplementary Information 4**

**Handling missing data**

***Missing data occurrence***

Missing data occurred for inpatient indicators in a given month for a given hospital.

Ownership (public or private), whether hospital belongs to a clinical information network (CIN), time (month and year) and COVID-19 binary indicator (0 – months before pandemic and 1 – months post pandemic) were used as covariates with the hospital ID as a clustering variable. The hospital characteristics were fully observed across all hospitals.

### **Multiple Imputation under joint modelling framework**

We implemented MI under the joint modelling imputation framework. In instances with complete data, standard statistical approaches for multi-level data apply models accounting for this dependency (1). Similarly, for missing values, imputation techniques need to account for dependency between observations, otherwise the predictive variance of the missing data is not accurately reflected. Certainly, if an incomplete variable is imputed ignoring the multilevel structure, the resultant imputations can be unreliable (1), and consequently bias in estimates obtained from imputed data. Therefore, to account for the multilevel structure, imputation techniques based on regression models that include a random intercept for clusters are generally used and have been implemented in the R-package *jomo* (2-4).

***General model specification under joint modelling framework***

Since missing data was observed in each inpatient indicator (outcome variable) in our dataset, with hospital characteristics fully observed (covariates), this presents a univariate missing data pattern (1). The general imputation model under this scenario is outlined below (1);

Let the matrix $Y_{n\times p}=\left( y_{1},\ldots.,y_{p} \right)$ be the matrix of incomplete data for $n$ items in rows and $p$ variables in columns. Let $i$ be the index for the individuals ($(1\leq i\leq n$ and $j$ for columns $(1\leq j\leq p)$. Y is stratified to K clusters of size $n_{k}$ where $k$ denotes the index for a cluster $(1\leq k\leq K)$. So, $y_{jk}$ denotes the $n_{k}$- vector corresponding to vector $y_{j}$ restricted to individuals within cluster $k.$ Then, let $(y_{j}^{obs},y_{j}^{miss})$ be the missing and observed parts of $y_{j}$ and let $Y^{obs}=\left( y_{1}^{obs},\ldots, y_{p}^{obs} \right)$ and $Y^{miss}=(y_{1}^{miss},\ldots, y_{p}^{miss}$). The imputation draws missing values from the predictive distribution $P(Y^{miss}|Y^{obs})$, where, an imputation model with parameter θ is specified and realizations of the predictive distribution of the missing values can be obtained by; drawing θ from $P\left( \theta| Y^{obs} \right)$ its posterior distribution and drawing missing values according to $P\left( Y^{miss} | Y^{obs},\theta\right)$ their predictive distribution given θ (1). In our case of a single incomplete variable $\left( y_{p} \right)$, the posterior distribution can be specified by letting $\theta=(\beta,Ѱ,\sum_{k})$ be the parameters of a linear mixed effects model (1):

|  | $y_{pk}=Z_{k}\beta+W_{k}b_{k}+\varepsilon_{k},$ | (1) |
| --- | --- | --- |

|  | $b_{k}\sim N\left( 0,Ѱ \right),$ | (2) |
| --- | --- | --- |

|  | $\varepsilon_{k}\sim N(0,\sum_{k})$ | (3) |
| --- | --- | --- |

Where $y_{pk}$ denotes the incomplete variable restricted to the cluster $k,$ $Z_{k}(n_{k}\times q)$ and $W_{k}(n_{k}\times q^{'})$ are the known covariate arrays corresponding to two subsets of $\left( y_{1k},\ldots,y\left( p-1 \right)k \right),$ β is the $q-$vector of regression coefficients of fixed effects, $b_{k}$ is the $q^{'}$-vector of random effects for cluster $k$, Ѱ$(q^{'}\times q^{'})$ is the between cluster variance matrix, and $\sum_{k}=\delta_{k}^{2}I_{nk}\left( n_{k}\times n_{k} \right)$ is the variance matrix within cluster $k$.

***Implementation of the model to DHIS2***

Since the number of admissions and deliveries is count, linear transformation was important before imputation, following an appropriate variance-stabilizing transformation to make the normal distribution assumption more plausible. The variance-stabilizing transformation for the Poisson distribution of count data is the square root, and it provides a better transformation relative to the log transformation for count data (5-9). The back transformed values under the square root method align with the original count scale. A linear mixed effects model was then selected for the implementation of MI. Below is a representation of the specified model in matrix form:

|  | $Y_{i}=X_{i}\beta+Z_{j}b_{j}+\varepsilon_{i}$ | (4) |
| --- | --- | --- |

Where: $Y_{i}$ is response vector of inpatient indicators (admissions and deliveries), $X_{i}$ the model matrix for the fixed effects (hospital covariates; ownership (whether hospital is public or private), CIN (whether hospital belongs to a CIN), COVID-19 binary indicator and time (time data was reported as months and year combined)) and $Z_{i}$ the model matrix for the random intercept for observations in the $j^{th}$ hospital. The vector of hospital covariates coefficients is represented by $\beta$ while $b_{j}$ represents the vector of random-effect coefficients in hospital $j$ . The errors terms denoted by $\epsilon_{i}$ are assumed to follow a multivariate normal distribution with mean vector 0 and variance covariance matrix ∑. The MI mixed effects model was implemented in R version 3.6.3 using the *jomo* package for multilevel imputation (10).

**References**

1. Audigier V, White IR, Jolani S, Debray TP, Quartagno M, Carpenter J, et al. Multiple imputation for multilevel data with continuous and binary variables. Statistical Science. 2018;33(2):160-83.

2. Quartagno M, Grund S, Carpenter J. Jomo: a flexible package for two-level joint modelling multiple imputation. R Journal. 2019;9(1).

3. Quartagno M, Carpenter J, Quartagno MM, BaBooN S. Package ‘jomo’. 2020.

4. Quartagno M, Carpenter J. Multiple imputation for IPD meta‐analysis: allowing for heterogeneity and studies with missing covariates. Stat Med. 2016;35(17):2938-54.

5. Quartagno M. Multiple Imputation for Individual Patient Data Meta-Analyses: London School of Hygiene & Tropical Medicine; 2016.

6. Yu G. Variance stabilizing transformations of Poisson, binomial and negative binomial distributions. Statistics & Probability Letters. 2009;79(14):1621-9.

7. Crawley MJ. The R book: John Wiley & Sons; 2012.

8. Maindonald J, Braun J. Data analysis and graphics using R: an example-based approach: Cambridge University Press; 2006.

9. Stroup WW. Rethinking the analysis of non‐normal data in plant and soil science. Agron J. 2015;107(2):811-27.

10. Quartagno M, Grund S, Carpenter J. Jomo: a flexible package for two-level joint modelling multiple imputation. R Journal. 2019.
