# Supplementary material for: The indirect impact of COVID-19 pandemic on inpatient admissions in 204 Kenyan hospitals: An interrupted time series analysis
Source: PLOS Glob Public Health. 2021 Nov 17;1(11):e0000029. doi: 10.1371/journal.pgph.0000029 (PMC10021711; doi:10.1371/journal.pgph.0000029)
Supplement: Attachment — Submitted filename: Response to Reviewers.docx [file pgph.0000029.s005.docx]

**Editor**

- Selection criteria for the hospitals/care facilities to include is quite important and would like more explicit language, if possible, in and around line 119 where a "considerable number" of inpatients justified inclusion

*We appreciate the comments made by the Editor and made efforts to address these. The hospitals included in this study were selected based on a criterion outlined by* ***Ouma et al 2020, Mapping first level referral hospitals in Kenya, during his PhD****, unpublished work. We have replaced “considerable number” with on average 5,408 admissions annually, used to define a hospital. Line 124-125. More details on the multiple datasets and other characteristics used to refine the definition of a hospital are provided in the paragraph “selection of hospitals”*

- Please provide additional detail/justification, if possible, for why the national healthcare worker strike was limited to the public sector facilities.

*The strike only occurred in the public hospitals, where health care workers demanded for better working conditions such as provision of adequate PPEs and better insurance. Since public and private health facilities are managed differently, channelling of resources for provision of such services is not uniform.*

*Although the government assured Kenyan health workers that there were enough PPE, most hospitals could not provide doctors with masks and personal protective equipment triggering the protest over gear shortages (*[*https://www.ncbi.nlm.nih.gov/pmc/articles/PMC7565744/*](https://www.ncbi.nlm.nih.gov/pmc/articles/PMC7565744/)*).*

*Information added in line 99-103*

**Reviewer's Responses to Questions**

**Reviewer 1**

This is an excellent paper on the indirect impact of COVID-19 pandemic on inpatient admissions in 204 Kenyan hospitals. The paper is well-written, and methodologically sound. The authors did not only use the DHIS2 data sets that have some weakness, but took care of the robustness and validity of the data by using other hospital data systems.

The stats are well elaborated; the result section provides interesting data of 5 key indicators of hospital admissions, and leads to important observations that are well supported and discussed in the discussion section. Limitations are described as well.

The authors have observed public and private hospitals and described delivery trends in covid times, taking into account also the effects of the health care workers strike- indicating the resilience of the maternal health care systems in Kenya. Obviously, more difficult to measure is the number of births at home or with TBAs, as the birth registration systems are incomplete in many parts of the country.

In conclusion: great work!

*We thank the Reviewer for recognizing the importance of this work. On the aspect of events occurring outside health facilities, we agree with the review that it is difficult to quantify number of births occurring outside the health system, we have added this as a possible limitation. Line 379-381. However in Kenya 61% of deliveries in 2014 occurred in the health facility with skilled attendance at delivery above 90% (Kenya Demographic Health Survey 2014). Based on historical trends our data suggests that the number of deliveries in hospital were unaffected.*

Reviewer #2: Line 54: the authors write: “The spread of the pandemic has placed unpresented challenge on health systems.” I presume they meant unprecedented

*We thank the Reviewer for taking the time to review the manuscript and for pointing out this error.*

*‘*unpresented” has been replaced with ‘unprecedented’c, line 54

What was the impact of the lockdowns? One could argue that the lockdown restricting mobility was the direct course as opposed to health facility stigma as vectors of transmission. This paper will improve dramatically if the authors include mobility data e.g. facebook/google mobility data for social good.

*We appreciate this comment however it is our opinion that restricted mobility is not the only factor to consider here and argue that it is a mix of various factors interplaying to affect the utilization of health services. These include fear of increased exposure in health facilities, reduced incomes affecting ability to meet transport costs and other healthcare related costs, shortage of healthcare workers in health facilities limiting appointments to the facilities, difficulties in accessing health facilities due to lockdowns and curfew and closing of some health facilities as documented in data from various surveys conducted in Kenya (*[*https://preventepidemics.org/wp-content/uploads/2020/11/PERC-Brief-Essential-Services_Report_1120.pdf*](https://preventepidemics.org/wp-content/uploads/2020/11/PERC-Brief-Essential-Services_Report_1120.pdf)*). Additionally, since the lockdown was not uniform across the country (for instance there are counties where lockdown hasn’t been instituted since start of the pandemic), it would be difficult to disentangle the singular impact of the lockdown in this national study, but it is a contributing factor to the changes alongside fear and others.*

*Further, we explored the COVID-19 stringency index for Kenya. (https://ourworldindata.org/grapher/covid-stringency-index?tab=chart&region=Africa&country=~KEN), which is a composite measure of indicators like school closure, travel bans and is an indirect measure of mobility. The index indicated that the lockdown measures were most stringent around April 2020 for Kenya, the interruption point in our study. More information about composition and calculation of the index can be found here (https://www.nature.com/articles/s41562-021-01079-8) This has been added in line 192-193 with references.*

Line 145-147: It is unclear how the authors implemented multiple imputation for missing data. Reviewers and the readership may benefit from a complete presentation of the algorithm used in the imputation process as supplementary material. What assumptions were made and whether sensitivity analyses for the missing data mechanism were performed. I am especially keen to see how the joint model was formulated /specified.

The authors mention the covariates used in the imputation model, but no information on which variables/indicators were imputed. The number imputations is also missing.

*We used the joint modelling approach, and used a multilevel imputation model, where data was imputed at the hospital level. We assumed the missing at random (MAR) assumption. We didn’t carry out sensitivity analysis, but this is work we are considering in the future to assess the departure from the MAR assumption. This has now been included as a limitation of this study line 384*

*We used the incomplete indicators (inpatient indicators) as outcomes and hospital characteristics and time as covariates. Each indicator was imputed separately. We used the* ***jomo*** *package and obtained 100 imputations for each indicator. 154-158*

*The imputation method has now been specified and explained in detail in the Supplementary File 4, Handling missing data, and referenced on line 166.*

Line 188: The authors say, “A generalized linear model was applied, assuming a Poisson distribution. We fitted both Poisson and negative binomial models to account for overdispersion (30-32)”. There is no reason why the authors are fitting both models unless it is at the point of model selection in which case only the optimum model results should be presented in the main results. Was the data over dispersed? Given the volume of the data, does the Poisson model approximate the normal distribution?

*As part of this analysis, we assessed both Poisson and Negative binomial models to select the optimum model (model selection). The statement indicating the final model has now been moved from “results section” to “methods section” in line 210-212 and clarified that the negative binomial model was the best model and we present estimates from the model in this paper. Yes, the count data was over dispersed, and the Poisson model wouldn’t be the best fit as it may not approximate the normal distribution given the volume of our data, and therefore the results discussed in this paper are for the negative binomial model*

Line 197 “a hospital level mixed-effects model using generalized estimating equations (GEE)” is incorrect. This is because mixed effects and marginal GEE are two distinct model families and parameter estimates one model family cannot be used to assess robustness of the other more so when dealing with non-continuous outcomes.

*We thank the reviewer for catching this error this has now been rectified and a rational provided in the manuscript which was to use it for sensitivity analysis. This is now included in the description of the model, Line 221-223*

*We used a Poisson GEE as a sensitivity model to test the effect of varying model assumptions, for instance any hospital-specific differences, on the estimates similar to Siedner et al 2020 (*[*https://bmjopen.bmj.com/content/10/10/e043763*](https://bmjopen.bmj.com/content/10/10/e043763) *). Reference has also been provided in line 223*

Hence Line 257: “The estimates obtained from hospital level generalized estimating equations (GEE) were not different from the primary model estimates hence robustness of the estimates.” could be misleading for the reason above

*We have corrected the aim of the Poisson GEE was to test the effect of varying model assumptions and not evaluate robustness of the estimates.*

Sensitivity analyses with the GEE model was performed to assess the potential variability by hospital/health facility. Please indicate the distribution of the error term (Normal, Poisson or negative binomial). Were some hospitals more affected by the pandemic compared to other? Explain the variability observed

*The distribution of the error term was Poisson. The reporting varied by hospital due to differences in infrastructural capabilities. It is possible the pandemic affected the hospitals differently, for instance some hospitals were assigned as isolation centres or closed for some time. We couldn’t obtain data on periods the specific hospitals were assigned as isolation centres or closed. In addition, the magnitude of the epidemic in some counties was higher. Therefore, in counties with higher case burden, some hospital staff were re-assigned or had to go for quarantine if they contracted COVID-19, leading to shortages. We have now added information that explains the variability in line 215-220.*

I would also expect the so see parameter estimates for the variance between hospitals

*We have added a table on intra-class correlation in Supplementary File 3 Table 3. Added to line 221*

The authors need to define RR when it is first used. Count models output the incidence rate ratios (IRR) and not the risk ratios (RR).

*To clarify we have used relative risk (RR) for CS and NBU rates where we used live births and total deliveries as* ***offsets****, to account for any differences in the denominators overtime (now added the word offset on line 183-184) as described by Bernal et al 2017 (https://pubmed.ncbi.nlm.nih.gov/27283160/).*

*For the other indicators we used admission counts without offsets hence we have corrected this to correctly reflect IRR.*

Caesarean section rate and NBU the authors considered proportions and but fitted Poisson and negative binomial. why didnt they consider beta regression model?

*This is related to the previous comment. To clarify, for caesarean sections and NBU admissions rates we used live births and total deliveries as* ***offsets*** *in the ITS model as described by Bernal et al 2017. Hence the use of Poisson and negative binomial distribution.*

Lines 231-232: “The negative binomial segmented ITS model, which accounted for seasonality, autocorrelation and overdispersion was the best model when compared with Poisson and negative binomial models that were not adjusted”. However, parameter estimates for the ARMA(p,q) part of the best fitting model are missing in the results section.

*Thanks, autocorrelation occurred in some indicators (admissions in Medical, Surgical and Paediatrics) and only for these indicators were the ARMA models fitted. We tried a combination of p and q and selected the combination that led to the lowest Akaike Information Criterion. Added in line 206-209*

*Therefore, in the results the estimates presented are those obtained from the model with lowest AIC. The combinations of p and q used have been added in the footnote of Table 1, line 590*

Line 283. sentence starting with “ the..

Thanks, updated to start with capital “The”
